# Work hours, weight status, and weight-related behaviors: a study of metro transit workers

**DOI:** 10.1186/1479-5868-7-91

**Published:** 2010-12-20

**Authors:** Kamisha H Escoto, Simone A French, Lisa J Harnack, Traci L Toomey, Peter J Hannan, Nathan R Mitchell

**Affiliations:** 1 University of Minnesota, Department of Psychiatry, 606 24th Avenue South, #602, Minneapolis, MN 55454, USA; 2 University of Minnesota, Division of Epidemiology & Community Health, Suite 300 WBOB, 1300 South Second Street, Minneapolis, MN 55454, USA

## Abstract

**Background:**

Associations between hours worked per week and Body Mass Index (BMI), food intake, physical activity, and perceptions of eating healthy at work were examined in a sample of transit workers.

**Methods:**

Survey data were collected from 1086 transit workers. Participants reported hours worked per week, food choices, leisure-time physical activity and perceptions of the work environment with regard to healthy eating. Height and weight were measured for each participant. Multivariate linear and logistic regressions were conducted to examine associations between work hours and behavioral variables. Associations were examined in the full sample and stratified by gender.

**Results:**

Transit workers working in the highest work hour categories had higher BMI and poorer dietary habits, with results differing by gender. Working 50 or more hours per week was associated with higher BMI among men but not women. Additionally, working 50 or more hours per week was significantly associated with higher frequency of accessing cold beverage, cold food, and snack vending machines among men. Working 40 or more hours per week was associated with higher frequency of accessing cold food vending machines among women. Reported frequency of fruit and vegetable intake was highest among women working 50 or more hours per week. Intake of sweets, sugar sweetened beverages, and fast food did not vary with work hours in men or women. Physical activity and perception of ease of eating healthy at work were not associated with work hours in men or women.

**Conclusions:**

Long work hours were associated with more frequent use of garage vending machines and higher BMI in transit workers, with associations found primarily among men. Long work hours may increase dependence upon food availability at the worksite, which highlights the importance of availability of healthy food choices.

## Background

Obesity rates are high among employed adults, have shown consistent increase over the past few decades and vary by occupational group [1]. Most adults spend 8 to 12 hours a day at work, which likely affects other domains in their lives such as self-care behavior and physical health [2,3]. It is therefore important to determine the aspects of employment that influence body weight and weight-related behaviors. The exact mechanisms that underlie the relationship between work and obesity are unclear, but may include physical and social aspects of the work environment and structural aspects of work, such as the number of hours worked per week. Given the potentially significant impact of work on obesity, understanding the nature of this relationship could inform the development of effective prevention and treatment interventions.

Previous research has shown inconsistent associations between the number of hours worked per week and weight, dietary intake, food habits, and physical activity, with associations often differing by gender. Much of the literature suggests that longer work hours are associated with higher Body Mass Index (BMI) [4-8], poorer dietary habits [9-12] and reduced leisure time physical activity [13-16], with associations occurring often among men and not women [5-7,13]. However not all research supports these findings; several studies report no association between hours worked per week and weight, food choice, or physical activity [3,7,17-19] or report associations only among women [8,9]. More research is needed to clarify the relationship between work hours, weight, and weight-related behaviors [11,20] and the potential gender differences in these associations [21]. In addition, few studies have examined these relationships within single occupational groups, which may be important in understanding work-related factors that impact weight in occupations with high obesity prevalence. Further, understanding the importance of work-related factors, such as work hours, may assist in the design of worksite interventions and policies addressing obesity [1,22].

The purpose of the current study was to examine associations between work hours, food intake, physical activity and perception of ability to eat healthy at work in a sample of transit workers, composed primarily of bus operators. In a study of major U.S. occupational groups, individuals employed as motor vehicle operators (which includes bus drivers) had the highest prevalence of obesity, with estimates at 31.7% for men and 31.0% for women [1]. In addition to high rates of obesity, transit workers suffer from stressful conditions of work. Along with being a largely sedentary job [23,24], structural characteristics such as shift patterns and work hours poses challenges for consistent engagement in healthy weight management behaviors [25]. Long and irregular hours are common, creating difficulties for family meals [23], constraining time available for meal preparation and exercise [22], and potentially limiting access to healthy foods [26]. While previous research has examined associations between long work hours and outcomes such as back and neck pain [27], occupational fatigue [28,29], and psychological health [30] in transportation workers little to no research has examined associations with weight and weight-related behaviors in this occupational group. The present study examined associations between work hours and behavioral variables and among men and women as prior research has identified gender differences between work hours, health behaviors and obesity [7,9,31]. It was hypothesized that that work hours would be associated positively with BMI, poorer dietary habits, lower physical activity, and greater perceived difficulty of eating healthy at work.

## Methods

### Sample and procedures

Data for this study were collected as part of a worksite environmental obesity prevention intervention among Metro Transit workers (the Route H study). Details of the study design are published elsewhere [32,33]. The study was a group-randomized trial conducted with four transit garages. Interventions components targeted the garage food and physical activity environment and included increasing availability of healthy foods at the worksite, improvements in the fitness facilities and increasing the availability of group-based programs to promote healthful eating and physical activity behaviors. Measurements were collected at baseline and after the 18-month intervention ended. Baseline measurements only were analyzed for the present study.

All employees at the four garages were invited to complete survey instruments and have height and weight measured by research staff. The average individual participation rate across the garages was 78% (69% to 84%). The sample for the present study was comprised of 1086 workers (854 men; 232 women) who completed the baseline survey and answered the work hours question. Participants received a $20 incentive for completing the survey and height and weight measurements.

This study was approved by the University of Minnesota Institutional Research Board Human Subjects Protection Program.

### Measures

#### Work Hours

Work hours were measured using a single item that queried the number of hours worked at Metro Transit per week on average. Response options were *0 - 10 hours; >10 - 19 hours; 20 - 29 hours; 30 - 39 hours; 40 - 49 hours; or ≥ 50 hours per week*. For analysis purposes, categories were collapsed into *< 40 hours*, *40 - 49 hours*, and *≥ 50 hours *per week.

#### Body Mass Index (BMI)

Body weight was measured in street clothing using an electronic, calibrated scale by trained research staff. Height was measured using a portable stadiometer. Two separate measurements were taken and the average of the two values were used to calculate BMI for analysis purposes. BMI was calculated as weight (kg)/height (m^2^).

#### Food Intake

Food intake was measured using a self-report food frequency questionnaire. The food frequency instrument measured a subset of foods targeted by the intervention and was adapted from two existing instruments with established validity [34]. Participants reported weekly frequency and serving size of foods consumed in the past month. For the current study, intake of fruits and vegetables (4 items), sweets (6 items), salty snacks (4 items) and sugar sweetened beverages (SSB; 2 items) was examined. Fruits and vegetable items were 100% juice, fruit, lettuce salad, and other vegetables (all raw and cooked). Sweet foods consisted of food items such as ice cream, cookies, doughnuts, sweet muffins and chocolate candy. Salty snacks were food items such as chips, popcorn, and french fries; SSB were defined as fruit drinks and soft drinks (regular, non-diet). Summary scores for food categories (e.g., sweets) were calculated by multiplying the frequency of consumption of each food item (e.g., chocolate candy) per week by the usual portion size, dividing by seven to yield portion size per day and summing across items. One item queried frequency of consuming breakfast, lunch, or dinner at fast food places (e.g., McDonald's, Kentucky Fried Chicken) over the past month. Response options were *never, 1-3 times last month, 1-2 times per week, 3-4 times per week, 5-6 times per week, and ≥7 times per week*. For analysis purposes, responses were categorized as *no fast food restaurant use*, or *≥ 1-3 times per month*.

As vending machines were the only source of food that could be purchased onsite at the garages [35], we assessed frequency of access to the four types of vending machines available: snack, cold food, hot beverage, and cold beverage. The vending machines consisted primarily of food items that were considered less healthful. Across the study garages, between 21% and 37% of the vending items met healthful criteria of being low calorie (i.e., ≤ 400 kcals for entrees; ≤ 150 kcals for snacks and sweets; ≤ 50 kcals for cold beverages; ≤ 120 kcals for milk), low sugar (i.e., ≤ 35% by weight for entrees, snacks, sweets; nuts, seeds, mints, and gum were sugar-free), and lowfat (i.e., ≤ 30% total kcals for snacks, entrees, sweets, cold beverages) and most items were high calorie [36]. Frequency was assessed during the past month, with response options being *never, 1-3 times last month, 1-2 times per week, 3-4 times per week, 5-6 times per week*, and *≥7 times per week*. For analysis purposes, responses were categorized as ≥ *3-4 times/week *vs. *1-2 times/week or less *for snack, hot and cold beverage machines, and *≥ 2-3 times/month *vs. *1 time/month or less *for cold food machines.

#### Physical Activity

A self-report measure was used to assess leisure time physical activity (LTPA) [37,38]. Participants reported the number of times per week they engaged in strenuous, moderate, and mild leisure time activity for more than 10 minutes. Additionally, participants reported their frequency of sweat-inducing exercise episodes during a 7-day period (never, sometimes, often). The number of moderate and strenuous episodes were combined and dichotomized into *≥ 3 times per week *vs. *<3 times per week*. The number of mild episodes was also dichotomized into *≥ 3 times per week *vs. *<3 times per week*. Finally, sweat-inducing exercise was dichotomized into *sometimes/often *vs. *never*.

#### Perceived worksite environment related to healthful food choices

Two single-item measures assessed perception of ease of eating healthy at work. Items were "At my workplace it is easy to eat a healthy diet" and "It's hard for me to get fruits and vegetables when I'm at work". The response scale was a five-point Likert scale ranging from *strongly agree *to *strongly disagree*. For analysis purposes, the items were recoded to a dichotomous response: *agree or strongly agree *vs. *neutral, disagree, or strongly disagree *(hard to get fruits and vegetables) and *disagree or disagree strongly *vs. *neutral, agree, or strongly agree *(easy to eat a healthy diet).

#### Demographics

Demographic variables were self-reported and included age, gender, race (coded white vs. other), education (coded high school or less, some college, college degree or higher), marital status (coded married or partnered vs. not), annual household income (coded as ≥$50001 vs. < $50001), years worked at Metro Transit (coded as up to 5 years, > 5 - 15 years, > 15 years), and job position (coded as bus operator vs. other). Smoking status was measured using three questions. Current smokers were those who reported having smoked at least 100 cigarettes in their lifetime *and *had smoked a cigarette in the past seven days.

### Statistical analysis

Analyses were conducted using SPSS Version 16.0 (SPSS for Windows, Rel. 16.0.1. 2007. Chicago: SPSS, Inc). Frequencies and means were calculated for each variable in the full sample. With the exception of age and BMI, all variables were coded as categorical due to their non-normal distributions. Chi-square analyses were conducted to examine bivariate associations between hours worked per week and the categorical sociodemographic variables. Comparisons of age differences by work hour category were examined using one-way ANOVA. Multivariate logistic regression analyses were conducted in the full sample and gender-stratified to examine associations between work hours and the dichotomized behavioral variables. Multivariate linear regression analyses were conducted to examine associations between work hours and BMI, the only continuous dependent variable. Models were first examined with adjustment for basic demographic variables age, gender, and race (Model 1). Then a second model was examined with full adjustment for sociodemographic and other work-related variables (job position, education, income, race, marital status, and tenure at Metro Transit). Control variables were selected because they were associated with hours worked per week in bivariate analyses (years worked at Metro Transit, job position) or have shown associations with weight status or behavioral variables in the literature [39-41].

Models were examined to determine if the overall work hour variable was statistically significant and p-values are presented for both Model 1 (base model) and Model 2 (fully adjusted model). Crude prevalence of transit workers reporting endorsement of the behavioral outcome variables are presented, with asterisks denoting significant differences (p < 0.05) in work hour categories from the reference category (< 40 hours per week) in the fully adjusted models. Due to the small number of women reporting use of snack and cold food vending machines in the 50 or more hour per week category, this category was combined with the 40-49 hours category for those analyses.

## Results

### Demographics, Work, and Eating and Physical Activity Behavior Variables

Demographic variables, by hours worked per week, are presented in Table 1. Most of the sample (72%) worked 40 - 49 hours per week. The sample was primarily male and nearly two-thirds were white. About half the sample had completed at least some college and just over half (58%) had an annual household income $50,001/year or greater. Thirty-eight percent of the sample had worked at Metro Transit between five and 15 years. Nearly three-quarters of the sample were bus operators. With the exception of age and smoking status, significant differences by work hour category were observed for all other sociodemographic and work-related variables. Of note, high percentages of women and non-whites worked less than 40 hours per week, as well as those working at Metro Transit less than five years.

**Table 1:** Demographic characteristics and health behaviors of transit workers by hours worked per week (n = 1086)

		Hours worked per week			
	Total	<40	40-49	≥50	p^1^
N (%)	1086	221 (20.3)	785 (72.3)	80 (7.4)	
Age (SD)	47.7 (10.2)	46.3 (12.2)	47.9 (9.6)	49 (9.3)	.102
Gender (%)					.002
-Male	78.6	70.1	80.6	82.5	
-Female	21.4	29.9	19.4	17.5	
Race (%)					<.001
-White	62.7	52.9	66.4	53.8	
-Other	37.3	47.1	33.6	46.2	
Education (%)					.015
-High school/vocational	49.0	41.1	51.4	47.5	
-Some college	37.5	42.9	36.6	31.2	
-College or higher	13.5	16	12	21.2	
Marital status (% married or partnered)	60.0	52.7	61.8	62.5	.048
Income (%)					<.001
-Less than $50001/yr	42.4	58.6	39.6	26.2	
-$50001 or greater/yr	57.6	41.4	60.4	73.8	
Years worked at Metro Transit (%)					<.001
-Up to 5 years	31.5	56.1	25.8	18.8	
->5 - 15 years	38.0	28.5	40.8	37.5	
->15 years	30.5	15.4	33.4	43.8	
Position (%)					<.001
-Bus operator	72.9	95.5	65.3	86.2	
-Other	27.1	4.5	34.7	13.8	
Current smoker (%)	25.4	24.5	25.8	24.7	.664

^1^P values from chi-square tests except for all variables except age (comparison of means using one-way ANOVA test)

Obesity prevalence was high in the full sample, over half were classified as obese (BMI ≥ 30 kg/m^2^) and average BMI was 32.3 kg/m^2 ^(Table 2). Over half of the sample reported eating a half serving or more of sweets per day, while less than half reported consuming that serving size of salty snacks (40%) and sugar sweetened beverages (SSB) (38%). Less than half (44%) of the sample consumed 2 or more servings of fruits and vegetables per day. Frequency of fast food consumption was modest; 77% of Metro transit workers reported eating at a fast food restaurant 1 - 3 times or more in the past month. Approximately one third of Metro Transit workers reported using work vending machines 3 or more times per week. Self-reported leisure-time physical activity was fairly high, over half of the transit workers reported engaging in mild or moderate/strenuous exercise 3 or more times per week and 75% reported engaging in physical activity long enough to work up a sweat sometimes/often in a 7-day period. Nearly 65% of the sample found it difficult to eat healthy at work and just over half agreed it was difficult to get fruits and vegetables at work.

**Table 2:** Crude prevalence and multivariate associations: BMI, food intake, physical activity, and perception of healthy eating at work by hours worked per week in transit workers

	Hours worked per week				Models, p^1^	
					1	2
	Total	<40 (ref)^2^	40-49	≥50	p	p
N		221	785	80		
BMI (kg/m^2^) ^3 ^(SD)	32.3 (7.3)	32.2 (8.5)	32.2 (6.8)	34.4 (8.9)*		
Food Frequency						
Past month, ≥0.5 servings/day, %						
-Sweets	56.4	52.0	57.2	60.0	.57	.47
-Salty snacks	40.1	33.5	41.3	47.5	.04	.08
-SSB	38.1	37.6	37.7	43.8	.49	.23
Past month, ≥ 2 servings/day, %						
-Fruits and Vegetables	43.8	43.4	43.7	46.2	.97	.87
Past month, ≥ 1-3 times/month, %						
-Fast food	77.1	77.3	77.5	72.5	.74	.81
Past month use of vending machines, ≥ 3-4 times/week, %						
-Cold Beverage	33.7	26.3	34.8*	43.0*	.005	.01
-Hot Beverage	31.7	27.4	31.7	43.0	.12	.07
-Snack	31.5	25.1	32.0	43.8*	.01	.009
Past month use of vending machines, ≥ 2-3 times/mo, %						
-Cold Food	29.5	24.1	29.9*	40.0*	.02	.004
						
Physical Activity						
Moderate/Strenuous LTPA (% reporting ≥3 times/wk)	57.8	58.5	57.6	57.7	.79	.66
Mild LTPA (% reporting ≥3 times/wk)	50.2	45.5	51.9	46.2	.39	.65
Sweat Frequency (% reporting sometimes/often within past 7 days)	75.5	75.1	75.3	78.5	.80	.70
						
Perception of Work Environment						
Easy to eat healthy at work (%Disagree/strongly)	64.7	62.3	65.7	62.5	.49	.19
Hard to get F&V at work (%Agree/strongly)	52.2	49.5	52.8	54.4	.66	.19

Model 1 is adjusted for gender, age and race

Model 2 is adjusted for gender, age, race, education, job position, years worked at MT, and marital status.

^1^p-values represent significance of the overall work hour variable from multivariate logistic regression analyses (df = 2)

^2^Reference category is < 40 hours per week

^3^Multiple linear regression analysis with work hours coded as dummy variables (<40 v. 40-49; <40 v. ≥50)

*Denotes significant differences (p < 0.05) in prevalence from reference category (< 40 hours per week) in fully adjusted model (Model 2)

### Work Hours, Food Intake and Perceived Work Environment

Table 2 presents average BMI and crude prevalence of transit workers reporting frequency of food intake, physical activity, and perceptions of the work environment by work hour categories in the full sample. Associations existed between the number of hours worked per week and BMI, frequency of use of cold beverage, snack vending, and cold food vending machines; p-values are presented for the fully adjusted models only. BMI was highest among those who worked 50 or more hours per week and was significantly higher than those working less than 40 hours per week (34.4 v. 32.2 kg/m^2^, p = 0.03). Compared to transit workers working less than 40 hours per week, those working 50 or more hours per week made more frequent purchases from vending machines: snack (44% v. 25%, p = 0.002), cold beverage (43% v. 26%, p = 0.004), and cold food (40% v. 24%, p = 0.001). Cold food (30% v. 24%, p = 0.03) and cold beverage (35% v. 26%, p = 0.03) vending machine use was more frequent among those working 40 - 49 hours per week compared to less than 40 hours per week.

Transit workers working 50 or more hours per week were more likely to have a higher intake of salty snacks (48% v. 34%, p = 0.03 in Model 1), but the overall work hour variable was not statistically significant after adjustment. None of the other food frequency variables, leisure- time physical activity, or perceived ease of eating healthy at work was associated with the number of hours worked per week.

### Gender differences in work hours, food intake and perceived work environment

Generally, BMI, food intake, and perceptions of the work environment differed by gender. Average BMI was higher among females than males (33.4 kg/m^2 ^v. 32.1 kg/m^2^, p = 0.03). Compared with men, women reported higher intake of fruits and vegetables (53% v. 41%, p = 0.001) and less frequent use of cold food (21% v. 32%, p = 0.001) and hot beverage vending machines (24% v. 34%, p = 0.004).

Table 3 presents average BMI and crude prevalence of transit workers reporting frequency of food intake and perceptions of the work environment by work hour categories, stratified by gender. Results among males were analogous to the results in the full sample. Average BMI was highest among those who worked 50 or more hours per week and remained significantly higher than those working less than 40 hours per week (34.3 kg/m^2 ^v. 31.4 kg/m^2^, p = 0.02) after full adjustment. Compared to working less than 40 hours per week, men working 50 or more hours per week more frequently used cold beverage (48% v. 26%, p = 0.001) and cold food (47% v. 28%, p < 0.001) vending machines. Further, males working 40 - 49 hours (33% v. 22%, p = 0.04) and 50 or more hours (52% v. 22%, p < 0.001) per week used snack vending machines more frequently. Similar to the full sample, salty snack intake was highest in the 50 or more hours per week category, but the work hour variable was not significant in the fully adjusted model. No other food frequency or physical activity variables were associated with number of hours worked per week. With the exception of fruit and vegetable intake and cold food vending machine use, associations were not found between work hours and BMI, food frequency, or physical activity variables among females. Fruit and vegetable intake was highest among females working 50 or more hours per week (86% v. 56%, p = 0.04) compared to those working less than 40 hours per week. When analyzing fruits and vegetable items individually (i.e., fruit juice, fruit, lettuce salad, other vegetables) consumption of 'fruit' and 'lettuce salad' showed significant associations with number of hours worked per week (data not shown). Frequency of use of cold food vending machines was higher among women working 40 or more hours per week (23% v. 15%, p = 0.04) compared to those working less than 40 hours per week.

**Table 3:** Crude prevalence and multivariate associations: BMI, food intake, physical activity, and perception of healthy eating at work by hours worked per week stratified by gender

	Hours worked per week									
	Males			Models, p^1^		Females			Models, p^1^	
	<40 (ref)^2^	40-49	≥50	1	2	<40 (ref)^2^	40-49	≥50	1	2
n	155	633	66			66	152	14		
BMI (kg/m^2^) ^3 ^(SD)	31.4 (7.5)	32 (6.8	34.3 (9.1)*			34.1 (10.2)	32.9 (6.8)	34.9 (7.9)		
Food Frequency										
Past month, ≥ 0.5 servings/day, %										
-Sweets	50.3	57.5	59.1	.56	.75	56.1	55.9	64.3	.79	.66
-Salty snacks	27.7	42.7	45.5	.005	.08	47.0	35.5	57.1	.18	.28
-SSB	36.1	39.5	39.3	.28	.25	40.9	30.3	35.7	.35	.45
Past month, ≥ 2 servings/day, %										
Fruits and Vegetables	38.1	42.5	37.9	.28	.36	56.1	48.7	85.7*	.02	.04
Past month, ≥1-3 time, %										
Fast food	73.4	77.5	72.7	.57	.66	86.7	77.6	71.4	.36	.25
Past month, use of vending machines, ≥ 3-4 times/wk, %										
-Cold Beverage	25.8	34.7	47.7*	.005	.004	27.4	35.1	21.4	.11	.11
-Hot Beverage	28.0	34.0	46.2	.08	.06	25.8	22.1	28.6	.56	.39
-Snack^4^	21.9	33.2*	51.5*	<0.001	<0.001	32.8	25.5		.51	.50
Past month, use of vending machines, ≥ 2-3 times/mo, %										
-Cold Food^4^	28.0	31.3	47.0*	.01	.001	14.5	22.8*		.07	.04
										
Physical Activity										
Reporting LTPA ≥3 times/wk, %										
-Mod/Strenuous	60.9	60.5	63.1	.96	.96	52.5	44.8	30.8	.29	.25
-Mild	47.7	53.6	50.8	.46	.88	40.3	44.9	23.1	.48	.45
Sweat Frequency within the past 7 days,%										
-Sometimes/often	76.0	76.5	83.3	.42	.39	73.0	70.3	53.8	.36	.23
										
Perception of Work Environment										
Easy to eat healthy at work (% Disagree/strongly)	60.4	65.0	60.6	.37	.21	66.7	68.5	71.4	.93	.81
Hard to get F&V at work (%Agree/strongly)	49.0	51.3	52.3	.66	.19	50.8	58.9	64.3	.68	.68

Model 1 is adjusted for age and race

Model 2 is adjusted for age, race, education, job position, years worked at MT, and marital status.

^1^p-values represent significance of the overall work hour variable from multivariate logistic regression analyses (df = 2)

^2^Reference category is <40 hours per week

^3^Multiple linear regression analysis with work hours coded as dummy variables (<40 v. 40-49; <40 v. ≥50)

^4^Reference category is < 40 hours per week; 40 - 49 and ≥50 hours per week category combined due to small number of women working ≥50 hours per week endorsing snack and cold food vending machine use

*Denotes significant differences (p < 0.05) in prevalence from reference category (< 40 hours per week) in fully adjusted model (Model 2)

## Discussion

The findings of this study showed that long work hours were associated with high BMI and less healthful food habits in male transit workers; to a lesser extent, both healthful and less healthful food habits were associated with long work hours in female transit workers. Males working 50 or more hours per week had higher BMI and reported greater use of cold beverage, snack, and cold food vending machines than those working less than 40 hours per week. These associations were present after adjustment for both sociodemographic and work-related factors that are likely to account for differences in food habits, such as age, race, education, income and job position. In contrast, the number of hours worked per week was not associated with BMI and showed very few associations with food habits among female transit workers. Women working at least 40 hours per week more frequently used cold food vending machines. Surprisingly, those working 50 or more hours per week were most likely to consume 2 or more daily servings of fruits and vegetables. These results may indicate that the length of the working day may increase reliance on foods that are available at workplace facilities, particularly among male transit workers.

Aspects of the current study findings are consistent with prior research. As in other studies, obesity prevalence was high in this group of transit workers [1,42,43] and we identified associations between work hours and BMI among men only [5-7]. Further, work hours were associated with eating behavior between men and women differently [9,10]. In the present study, men working longer hours were more heavily dependent than were women on convenience foods purchased from the garage vending machines. Previous research indicates that men are more likely than women to purchase lunch at work and are more likely to report convenience as influencing food choice for lunch [44]. The gender difference in the association between work hours and vending machine use may be due to job position of the transit workers. A higher percentage of females were bus operators (85% vs. 70%, p < 0.001), thus may have had limited exposure to garage vending machines as their time at the worksite may be brief. Longer work hours, particularly for roles that might require more time at the garage (i.e., bus maintenance, management) may create opportunity for frequent use of onsite food sources. Female transit workers generally used vending machines less frequently than males (i.e., cold food, hot beverage).

Contrary to our expectations, female transit workers who worked 50 or more hours per were more likely to consume 2 or more servings of fruit and vegetables per day compared to those working less than 40 hours per week. The reasons for this association are not clear. Poorer dietary intake has been associated with longer work hours among women [9] and sociodemographic variables which may have helped explain the association (e.g., education, income, marital status) [45-48] did not vary significantly across work hour categories among females in our sample. Income was the only sociodemographic variable that was significantly associated with fruit and vegetable consumption; women having a household income of $50001 or more were more likely to report consuming 2 or more servings of fruits and vegetables per day (data not shown). The association between work hours and fruit and vegetable consumption may be due to obesity prevalence in females working 50 or more hours per week. This group of transit workers had the highest average BMI (34.9 kg/m^2^) in the sample, thus are likely to be attempting to lose weight [49]. Such individuals (i.e., overweight, obese) have been found to report higher consumption of fruits and vegetables [50] perhaps accounting for the prevalence in this work hour category. Finally, our finding may be due to the small number of women in this highest work hour category (≥ 50 hours per week). While 86% of women reported consuming 2 or more daily servings of fruits and vegetables, this represented 12 of 14 women. Hence, our findings need replication with a larger sample of female transit workers.

While the current study results indicate that transit workers who work long hours more frequently purchase foods from vending machines, other measures of poor eating behavior were not associated with hours worked, such as consuming fast foods, sweet foods, and sugar sweetened beverages. Further, work hours were not associated with reported leisure-time physical activity, which differs from the results of previous studies [14-16,51]. Although the relationship between hours worked per week and exercise has not been established consistently [18], bus operators report that more leisure time exercise and shortening the length of work days as among the most important ways to improve their health and work environment [52]. Additional research is needed to clarify the specific weight-related behaviors that are most disrupted by long work hours in transit workers. Future studies should examine variables such as meal frequency and meal timing, which have been associated with overweight and obesity [53]. As BMI was significantly higher among women than men, and over 60% of female transit workers were obese, further work should also focus on weight management in this population.

The findings of this study have implications for worksite intervention design and policy. Our results underscore the importance of worksite food environments containing healthy food choices that are readily accessible. In the Route H study, none of the garages had cafeterias onsite and only one garage had restaurants in the immediate area [36]. Thus vending machines were largely the only source of food available [35]. Transit workers who worked longer hours may have used vending machines regularly due to lack of other alternatives and/or time, given bus operators do not get scheduled meal breaks. Prior research suggests that workers frequently consume food from worksite facilities and that food offerings at the worksite are related to diet quality [54]. The limited food environment coupled with long working hours may be important contributing factors in the development and maintenance of obesity. Finally, in order to inform policy interventions, more research is needed to explore strategies such as flexible work arrangements and expanding driver control over work scheduling [23,55], given that our data suggests that long work hours carry consequences for weight and weight-related behavior in this occupational group.

The present study had several strengths and limitations. Strengths include a large mixed-gender sample of transit workers and several measures of dietary intake, eating behavior, and leisure time physical activity. This study is limited in its use of a cross-sectional design; no inferences can be made regarding causality. Additionally, our primary measures of dietary intake and physical activity were self-report, which may have been inaccurately reported [56,57]. The small sample of women may have limited our power in testing the gender-stratified associations. Only 6% of female transit workers were in the highest work hour category, potentially providing insufficient power to detect associations. Lower number of women working overtime has been found in other studies [58]. The current study's sample consisted primarily of bus operators (72%), but included small numbers of management and bus maintenance staff. Thus results reflect associations for transit workers across job positions, thus may not reflect the experience of solely bus operators. Our research contains a single work-related factor, the number of hours worked per week. Future research should expand measurement of work-related factors by including variables such as work shift, job stress, and job flexibility, which may be associated with health related behavior [22]. Finally, obesity prevalence was high in this sample, 56% of the transit workers were considered obese (BMI ≥ 30 kg/m^2^). Although high obesity prevalence is to be expected in a sample of transportation workers [1], this high prevalence may impact dietary and physical activity behaviors [49,50]. As the survey response rate was high in this study (78%), results may generalize well to transportation workers, but may have limited generalizability outside this occupational group.

## Conclusion

Longer work hours were associated with BMI, fruit and vegetable intake, and frequent use of garage vending machines in a sample of Metro Transit workers. Males who worked the longest hours (50 or more hours per week) had the highest BMI and were the most frequent users of vending machines at garage worksites. Longer work hours were associated with increased fruit and vegetable intake among women as well as more frequent use of cold food vending machines. Long work hours may increase dependence upon food availability at the worksite among transit workers, underscoring the importance of providing healthy food choices at the transit garages.

## Competing interests

The authors declare that they have no competing interests.

## Authors' contributions

SAF, LJF, TT and PJH contributed to the design of the study. KHE formulated the research question, conducted data analysis, and wrote the manuscript. PJH provided guidance with the statistical analysis of the data. All authors assisted with revising the manuscript. All authors read and approved the final manuscript.
